# Quality and Reliability of Halitosis Videos on YouTube as a Source of Information

**DOI:** 10.3390/dj9100120

**Published:** 2021-10-15

**Authors:** Atik Ramadhani, Zenobia Zettira, Yuanita Lely Rachmawati, Ninuk Hariyani, Diah Ayu Maharani

**Affiliations:** 1 Department of Preventive and Public Health Dentistry, Faculty of Dentistry, Universitas Indonesia, Jakarta 10430, Indonesia; atikramadhani@ui.ac.id (A.R.); zenobia.zettira@ui.ac.id (Z.Z.); 2 Department of Community and Preventive Dentistry, Faculty of Dentistry, Universitas Brawijaya, Malang 65145, Indonesia; yuanita.rachmawati@gmail.com; 3 Department of Dental Public Health, Faculty of Dental Medicine, Universitas Airlangga, Surabaya 60132, Indonesia; ninuk_hariyani@yahoo.co.id

**Keywords:** halitosis, videos, YouTube

## Abstract

Halitosis is a common condition that adversely affects quality of life. Through YouTube, people access oral health information. This study aimed to analyze the quality and comprehensiveness of the content, and reliability of halitosis videos hosted on YouTube. In total, 300 videos were collected based on three search keywords, and the number of likes, dislikes, views, duration, interaction index, viewing rate, and source category. Subsequently, the video score, which represents the content quality and comprehensiveness was used to categorize videos as poor, good, and excellent. DISCERN score was used to assess video reliability. Of the 105 videos analyzed, 68 (64.8%) were uploaded by personal users. In general, videos were categorized as poor and have low reliability. Videos sourced from healthcare professionals showed the highest quality, comprehensiveness of the content, and reliability (*p* < 0.05). There was no difference in the number of viewers for video duration less than or more than 4 min (*p* > 0.05). YouTube users prefer low-quality videos over high-quality ones, indicating that viewers cannot differentiate between reliable and potentially biased content. It is critical to enable viewers to critically assimilate information hosted on YouTube to make effective oral healthcare decisions.

## 1. Introduction

Halitosis is a common health problem worldwide that is found in all age groups [1]. Halitosis is the third most frequent complaint in patients, after cavities and periodontal disease [2]. The universally reported prevalence of halitosis varies widely, ranging from 22% to 50%. Contrasting results have been obtained in subjective (self-perceived) and objective examinations [3]. Several countries in Asia, such as Thailand, represent a pervasiveness of halitosis of 61.1%, and populations in China in the age range of 18 to 71 years show a prevalence of 65.9% [4].

The problem of halitosis has a complex etiology, but it is often ignored by society. This is because many people who are unaware that they have halitosis or feel ashamed of informing their condition to health professionals [2,5]. Halitosis may also be a manifestation of previously undetected systemic diseases. It can affect a person’s self-esteem, which has a negative impact on social interactions and a person’s quality of life. Social and psychosocial disorders, such as self-discomfort and bashfulness, are problems faced by halitosis sufferers, and are reasons for individuals to seek help [1,2].

Low self-perception and limited knowledge about halitosis are critical problems faced by sufferers [6]. Therefore, appropriate education is needed regarding the causes of halitosis and how to handle it. Providing video-based education is considered to be easier to receive, specifically for groups with low literacy levels. In addition, the videos available online can also reach a wider audience [7]. The emergence of digital technology has also influenced the role of early adopters in spreading messages online through video uploads [8]. In today’s digital era, the Internet has become a part of people’s lives in meeting information needs. Its rapid spread has made many people turn to the Internet as their first source of health information before seeking help from health professionals [9]. It is known that more than 70% of Internet users search for health information online [10]. The Internet as a health information-seeking platform assists a person in making healthcare decisions [11].

The need for communication through messages and social media is the key reason for many to use the Internet. Social media supports the process of seeking insight related to health, medication, medical care, sharing disease experiences among personal users, and communicating with health professionals or other patients [10]. Among the many social media platforms, YouTube is the most popular video-sharing site in the world, and more than two billion videos are watched per day. In Indonesia, the enthusiasm of Internet users for YouTube ranks high among the most visited sites by adolescents for oral health information [12]. The use of YouTube as a platform for health promotion and education can create interactions between users through likes, dislikes, comments, sharing videos, uploading videos, and more. YouTube is considered more efficient in enhancing patient understanding of disease risk, such as cancer, compared to other sites (Facebook, Twitter, and blogs) [13,14]. Despite its advantages, not many people recognize that the level of accuracy and reliability of YouTube videos is questionable [13]. Online video content is often found to be biased, contrary to evidence-based ones [15]. YouTube videos as a source of information on oral health requires further exploration. This study aimed to analyze the quality, comprehensiveness, and reliability of YouTube videos regarding halitosis.

## 2. Materials and Methods

The study was approved by the Ethics Committee of the Faculty of Dentistry, Universitas Indonesia, No. 18/Ethical Approval/FKGUI/VIII/2020 with Protocol No. 090190720. This study followed the PRISMA flow diagram for the video selection process [16,17]. YouTube was accessed and the keywords “bad breath”, “unpleasant breath”, and “oral malodor” were searched. Sort by relevance, a filter on YouTube, was used [18]. For each search keyword, 100 videos were selected and a total of 300 videos were analyzed further. The exclusion criteria included duplicate videos, videos that were not relevant to the search topic, and advertisements.

YouTube creates a variety of interactions between its users, which is reflected in the number of likes, dislikes, comments, sharing videos, uploading videos, and more [13,14]. There are ways to calculate the YouTube user interaction index rating, namely ([number of likes − number of dislikes/number of views] × 100) and viewing rate, which is measured by the formula ([number of views/number of days counted since the video was uploaded] × 100) [18]. In addition, the present study also collected data about video uploaders. This data were categorized as: (1) a healthcare professional, when the uploader was a health service provider (either on their behalf, in the presentation, or stated in the description), for example, a dentist; (2) non-professional, when the uploader’s qualifications was not known with certainty, for instance, a patient; (3) education channel, when information was only written or explained via voice messages; and (4) for-profit companies, when video uploaders promoted alternative products and claimed the products being effective under various conditions [19,20].

The quality of YouTube video information was assessed using the Global Quality Scale (GQS), which is based on the quality of information available, as well as assessing the extent to which the observed video is useful [21]. The GQS score is a 5-point Likert scale that explains the quality, flow, availability of information, and usefulness of information, with a maximum point for the best criteria [13]. In evaluating the comprehensiveness of the video content, the assessment was based on seven significant topic domains related to disease, including risk factors, epidemiology, causes, classification, diagnosis, management, and prevention of halitosis. Each topic was given a score of 0 to 2 (0 = not explained, 1 = explained briefly, and 2 = explained in detail), and the total score for the seven topic domains was 14 [22,23].

The GQS and comprehensiveness scores were then combined to obtain a video score that categorized the videos into poor with scores of 1–6, moderate scores of 7–13, and excellent scores of 14–19 [22,23]. The reliability of a video was assessed using DISCERN score [20]. For each aspect addressed, videos received 1 point, with possible scores ranging from 0 to 5 points. The criteria used in this analysis were as follows: (a) Are objectives clear and achieved? (b) Are the sources of information used reliable? (c) Is the information presented balanced and unbiased? (d) Are additional sources of information listed for patient reference? (e) Are areas of uncertainty mentioned? [18,21].

Data analyses were performed using the SPSS software (IBM SPSS 25.0; Armonk, NY, USA: IBM Corp). The interclass correlation coefficient (ICC) was used to specify the level of agreement between the two observers. The Kruskal–Wallis test was used to examine the differences in the characteristics of the video variables in the video source category, the video score category, and the DISCERN score category. Moreover, the Mann–Whitney test was carried out to determine the differences in the characteristics of the video variables on the video duration. Statistical analyses were based on the data distribution assumptions for numerical outcomes, purpose of test whether to examine the difference between two or more groups, or to measure strength of associations between variables [24]. Statistical significance was determined as a *p*-value < 0.05.

## 3. Results

**Figure 1:**
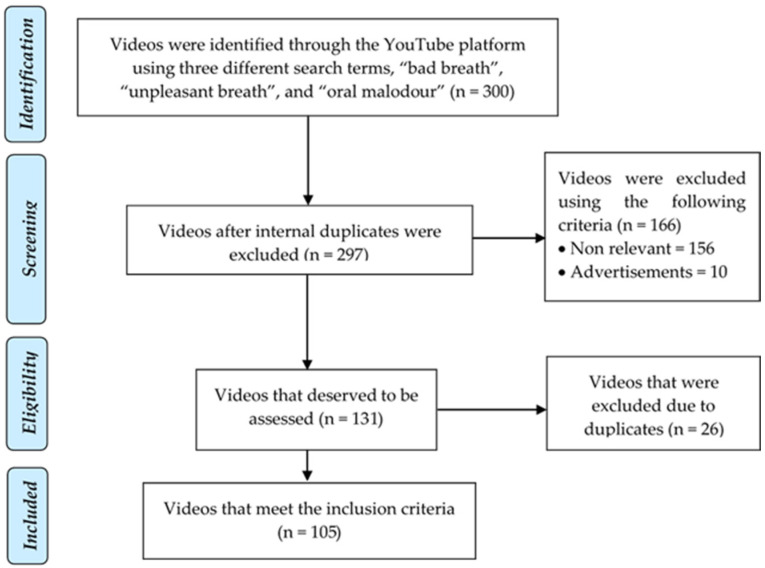
Selection of YouTube videos based on the PRISMA flow diagrams.

The inter-observer and intra-observer agreements on video score and DISCERN was good, with values ranging from 0.943 to 0.981. A search of three terms resulted in a total of 300 videos, of which 195 videos were excluded based on the exclusion criteria, and 105 videos were successfully obtained for further analysis (Figure 1). Table 1 shows that the most popular videos received 5255 likes, and the unpopular videos received 488 dislikes. In terms of views, the videos with the fewest views were watched 12 times, and those with the most views were watched 596,755 times. The medium length of the video was 4 min 53 s, starting with a video spanning 51 s to 13 min 19 s. Most of the videos observed (*n* = 68, 64.8%) were uploaded by non-professionals.

Table 2 shows the scores of the GQS, the comprehensiveness of the content discussed in the video, and the total video score. In terms of quality, most of the videos were of low quality and flow of explanation with some information available; however, not many important topics were discussed, and information was slightly useful (*n* = 48, 45.7%). Regarding the comprehensiveness of the video, in the majority, the following contents were not explained in the video: epidemiology, classification, and diagnosis. Meanwhile, content regarding management, prevention, and etiology in general, was only discussed with a brief explanation. According to the total score, based on the 105 videos evaluated, only a few (*n* = 8, 7.6%) videos were of the “excellent” category and most of them (*n* = 58, 55.2%) were included in the “poor” category.

Table 3 shows the results of the Kruskal–Wallis test, illustrating the comparison of video characteristic variables based on video source categories. There were significant differences between the uploader source groups in GQS, comprehensiveness score, total video score, and the DISCERN score (*p* < 0.05). Table 4 shows the results of the Kruskal–Wallis test, illustrating the comparison of video characteristics variables based on video scores which are categorized into “poor”, “good”, and “excellent”. The results of the statistical analysis showed that there was no significant difference between the video score category with likes, dislike, views, and viewing rates (*p* > 0.05). There were two variables that showed a statistically significant difference between the video score categories, namely, the interaction index and the DISCERN score. Post hoc analysis test was conducted if Kruskal Wallis test showed *p*-value < 0.05 (Table 3 and Table 4). Statistically significance between groups were apparent except comprehensiveness score, video score, and DISCERN score between non-professional and for-profit companies’ category. DISCERN score between health professional and education channel groups; interaction index between poor and good, and good and excellent category were also no statistically significant difference. Table 5 shows that there was a statistically significant difference in the interaction index based on the DISCERN value. The higher the interaction index, higher the number of likes, but fewer were the views. Videos with high-reliability scores (DISCERN) were found to have an increase in the number of likes faster than videos with low-reliability scores.

Duration is an aspect of the video that plays an important role in determining the level of the viewer’s attention. On the YouTube platform, there is a filter for video duration that can be selected by users, namely videos that are less than 4 min and more than 4 min. Videos that are less than 4 min tend to be concise and captivate the attention of a wider audience, while videos that are longer than 4 min tend to have a higher content discussion [25]. Table 6 highlights that four variables had significant differences between videos with durations of less than 4 min and more than 4 min, namely the interaction index, GQS score, video score, and the DISCERN score. The data show that the higher the interaction index value, the lower the number of viewers for videos with a duration of more than 4 min. The quality and reliability of videos are better for videos with a duration of more than 4 min. Videos that are less than 4 min tend to be watched a lot; nevertheless, the video quality was poorer. In addition to the duration filters available on the YouTube platform, it was found that in a learning video, it is crucial to maximize the viewer’s attention with the use of a shorter duration. The longer is the video duration, the lesser is the viewers’ attention.

## 4. Discussion

Halitosis is a dental and oral health problem that affects the general population worldwide. This multifactorial condition is known to make sufferers vulnerable to anxiety and shame (psychosocial burdens) that have an impact by decreasing a person’s quality of life. Following the Health Belief Model, a person will make health-related decisions when faced with psychological conditions, such as feeling vulnerable to disease [11]. The use of YouTube as a medium for health promotion and education can create various interactions between users through its characteristics such as likes, dislikes, views, sharing videos, uploading videos, and others [14]. In this study, 64.8% of videos about halitosis on YouTube were uploaded by non-professionals. This shows that the video uses language that is easily perceived by viewers with non-medical backgrounds and is patient-friendly. Videos that describe someone’s personal experiences are aired frequently, given that viewers find these types of videos more appealing than educational videos [13]. This contrasts with previous research, which states that as much as 50.9% of videos are uploaded by education channels and 60% of videos are uploaded by healthcare professionals [19,26].

Most videos assessed in this study described etiology, management, and halitosis prevention. However, overall, these videos do not discuss risk factors, epidemiology, classification, and diagnosis. It is arduous to expect every video to have a comprehensively structured discussion. Even though the content described is incomplete, some videos contain pivotal and accurate evidence-based information [27]. More than half of the YouTube videos studied were categorized as poor videos on being classified as poor, good, and excellent. This is probably because the majority of videos about halitosis were uploaded by non-professional users who are not medical personnel; thus, they have limited medical knowledge in delivering content. Most Internet sites related to halitosis also contain low-quality content that is generally confusing to users and leads to poor decision making [28]. This corresponds with previous studies that YouTube users tend to choose low-quality videos over high-quality ones [29]. Ordinary people might find educational videos less engaging; therefore, they tend to look for videos with alternative content that are often presented as low-quality videos. The video reliability based on the DISCERN rating resulted in low scores. The viewer received a lot of biased information and did not have a good understanding of the halitosis conditions they were experiencing. Hence, the viewer ignores the need to make a proper diagnosis considering that it is not supported by evidence-based information [28].

In addition, another important aspect showed that videos uploaded by healthcare professionals demonstrate a higher quality of information, comprehensiveness of content, and reliability [13]. Interestingly, the highest number of likes was found for poor-quality videos. Although it is not clear why viewers tend to like poor-quality videos over good-quality videos, the video duration could influence their choice. On average, good quality and reliable videos have a longer span but reduce viewers’ attention [29].

In the meantime, lower-quality videos are often uploaded by non-professional users. Videos with low quality are also more frequently watched by the public because viewers tend to follow suggestions by looking for other people’s personal experiences related to the disease they have experienced [29]. Other studies have revealed that videos uploaded by dental professionals are much more comprehensive than those uploaded by non-experts. It shows that they were made to educate the viewers [27]. Videos that are sourced from not-for-profit and for-profit companies lack quality, content comprehensiveness, and reliability since many videos were found that do not include reliable sources or additional references to viewers about bad breath. A previous study reported that incomplete information occurs in many unreliable video uploads, such as offering alternative therapies that have not been clinically tested [21]. Some video sources, particularly those from non-professionals, base their experiences or testimonies on alternative therapies such as herbal ingredients without explaining the side effects. Likewise, the video source was found to originate from therapeutic advertisements promoting their product findings, promising a speedy cure [29]. Videos sourced from health workers are found to have high-reliability compared to those that focus on the reliability of the treatment information provided [30].

Based on the video duration analysis, there was no difference in the number of viewers for videos less than 4 min and more than 4 min, both of which did not reduce the level of the viewers’ attention. However, the quality and reliability of videos are better for videos that are longer than 4 min. As a result, videos that are more than 4 min are considered efficient to educate viewers. A previous study revealed that videos that are less than 6 min are watched more, and videos with a time scale exceeding 6 to 9 min reduce the viewers’ attention [31]. Previous studies show that videos that are beneficial to the viewer are approximately 7 min in duration. This indicates that the video duration must be long enough for the content to be fully covered, but optimum video duration must be considered to avoid losing the viewers’ attention [18]. If the duration is too long, it may not engage viewers who are looking for a quick answer [29].

Video sources that come from non-professionals, such as influencers, have the largest number of viewers. Based on the innovation diffusion theory, influencers as video uploaders on YouTube are early adopters who are influential in spreading opinions among other adopters [8]. They follow the latest developments in spreading messages on social media. The ability of influencers to sway the attitudes, perceptions, and behavior of viewers makes them play a big role in the viewer’s eyes. Information conveyed by influencers in prompting the community has the potential to go viral because of their high level of popularity. The informal conversation style gives off a distinct impression of being handily accepted by viewers, consequently increasing the viewers’ attention [8].

The limitation in this study was that the search results for videos on YouTube were dynamic, which was presumed to change when new videos were uploaded, and old videos were deleted. During video searches, the results varied according to the date and time of the search. Multiple viewers may use different search terms, and it is possible to obtain mixed video results. YouTube does not facilitate ranking according to the high and low quality of the information contained in the video. This makes it difficult for viewers to obtain videos of good quality, trustworthiness, and those that are worth watching. There is no specifically validated scoring system available to assess the quality and reliability of videos before uploading them on YouTube. Researchers conducted observations and assessments independently, which allowed potential bias when making the assessment, but it can be overcome by conducting an inter-observer test between two observers calculated based on the ICC and producing good agreement on the assessment. Additionally, although bad breath is a lay term for halitosis [32], further study shall include halitosis as one of the search keywords, which may generate more results.

Evaluation criteria are needed regarding the quality of the video that is uploaded by each video uploader about halitosis on YouTube, with the hope that viewers can receive useful information, good quality content, and thereby, YouTube can be used as a reliable source of information on halitosis. The strategy for creating an effective health education video is to pay attention to the length of the video so that it is of a span of 4 to 6 min so that it includes comprehensive content without losing the viewers’ attention. Given a large number of poor-quality videos on halitosis, health workers and educational institutions play a fundamental role in improving the quality, comprehensiveness of the content, and reliability of this information to increase viewer eHealth literacy. Likewise, collaborations can be made between individual uploaders or influencers and health workers to reach a vast target audience. This collaboration is expected so that messages or health information can be transferred well and attention can be drawn. It is necessary to include additional references in YouTube videos to ensure that the video is not potentially biased. From this study, it can be concluded that, in general, YouTube videos about halitosis may be categorized as poor according to the content’s comprehensiveness and quality. YouTube users prefer low-quality videos over high-quality ones, which illustrates that viewers cannot differentiate between reliable content and potentially biased content. There is a need to enable viewers to critically assimilate information hosted on YouTube to be able to make effective healthcare decisions. Viewers need to be able to discriminate videos they view.

## Figures and Tables

**Table 1:** Characteristics of research videos.

Variables	Median (Min–Max)
Likes	21 (1–5255)
Dislikes	0 (0–488)
Views	686 (12–596,755)
Duration (mins)	04:53 (00:51–13:19)
Timespan since the video was uploaded (days)	182 (7–344)
Interaction Index	2.3 (0.1–1596.8)
Viewing Rate	450.7 (5.9–375,317.6)
GQS Score	2 (1–5)
Comprehensiveness Score	4 (1–11)
DISCERN Score	1 (1–5)

GQS: global quality scale.

**Table 2:** Frequency and percentage of the global quality scale score, the comprehensiveness score, and the total video score.

Global Quality Scale Score			
Item Scored			*n* (%)
The quality and flow of explanation is poor, information is inadequate; pointless information			5 (4.8%)
The quality and flow of explanation is low, some information is available but does not include many imperative topics; slightly beneficial information			48 (45.7%)
The quality and flow of explanation is moderate, some important information is available but others are poorly discussed; useful information			21 (20%)
The quality and flow of explanation is generally good, most of the relevant information is available but some are not covered; helpful information			18 (17.1%)
The quality and flow of explanation is exceptional, all relevant information is available; effective information			13 (12.4%)
Comprehensiveness Score			
Item scored	Not explained *n* (%)	Explained briefly *n* (%)	Explained in detail *n* (%)
Risk Factor	57 (54.3%)	22 (21%)	26 (24.8%)
Epidemiology	104 (99%)	1 (1%)	0 (0%)
Etiology	33 (31.4%)	40 (38.1%)	32 (30.5%)
Classification	97 (92.4%)	4 (3.8%)	4 (3.8%)
Diagnosis	97 (92.4%)	8 (7.6%)	0 (0%)
Management	17 (16.2%)	62 (59%)	26 (24.8%)
Prevention	6 (5.7%)	62 (59%)	37 (35.2%)
Total video score			
Video Score			N (%)
Poor (1–6)			58 (55.2%)
Good (7–13)			39 (37.1%)
Excellent (14–19)			8 (7.6%)

**Table 3:** Analysis of video variables based on video sources.

	Health Professional (*n* = 22)	Non-Professional (*n* = 68)	Education Channel (*n* = 10)	For-Profit Companies (*n* = 5)	*p*-Value
	Median (Min–Max)	Median (Min–Max)	Median (Min–Max)	Median (Min–Max)	
Likes	27 (6–383)	21 (1–5255)	16 (3–65)	25 (13–1164)	0.333
Dislikes	0 (0–82)	1 (0–488)	0 (0–18)	0 (0–45)	0.439
Views	583 (60–56,842)	982 (12–596,755)	307 (74–10,463)	649 (70–26,922)	0.186
Interaction Index	5.7 (0.5–21.7)	1.9 (0.4–32.5)	5.1 (0.1–12.6)	1.8 (0.9–1596.8)	0.062
Viewing Rate	434 (56.1–35,305.6)	514.7 (5.9–375,317.6)	339.3 (36.7–3180.2)	220 (21.1–11,407.6)	0.667
GQS score	5 (3–5)	2 (1–4)	4 (2–4)	2 (1–3)	0.001 *
Comprehensiveness score	8 (4–11)	3 (1–7)	6 (2–8)	2 (1–5)	0.001 *
Video score	13 (7–16)	5 (2–11)	10 (4–12)	4 (2–8)	0.001 *
DISCERN score	4 (3–5)	1 (1–4)	3 (1–5)	1 (1–3)	0.001 *

GOS, global quality scale; Kruskal–Wallis test; * *p* value < 0.05.

**Table 4:** Analysis of video variables based on the total video score.

	Poor (*n* = 58)	Good (*n* = 39)	Excellent (*n* = 8)	*p*-Value
	Median (Min–Max)	Median (Min–Max)	Median (Min–Max)	
Likes	23 (1–5255)	21 (2–2359)	19 (13–103)	0.958
Dislikes	0 (0–488)	0 (0–82)	0 (0–1)	0.112
Views	1130 (12–596,755)	493 (37–73,093)	437 (175–1.811)	0.144
Interaction Index	1.7 (0.4–1596.8)	3.4 (0.1–25.1)	7 (0.7–16.8)	0.029 *
Viewing Rate	613.3 (5.9–375,317.6)	292.5 (20–35,305.6)	583.5 (109.2–4100)	0.688
DISCERN score	1 (1–2)	3 (1–5)	4 (3–5)	0.001 *

Kruskal–Wallis test; * *p* value < 0.05.

**Table 5:** Comparison of popularity and visibility based on reliability.

	DISCERN Score (0–1) (*n* = 55)	DISCERN Score (2–5) (*n* = 50)	*p*-Value
	Median (Min–Max)	Median (Min–Max)	
Likes	20 (1–5255)	23 (3–2359)	0.409
Views	995 (12–596,755)	545 (37–73,093)	0.239
Interaction index	1.7 (0.4–1596.8)	3.7 (0.1–28.9)	0.008 *
Viewing rate	555 (5.9–375,317.6)	352.3 (20–35,305.6)	0.691

Mann–Whitney test; * *p*-value < 0.05.

**Table 6:** Comparison of video variables based on duration.

	Less than 4 min (*n* = 41)	More than 4 min (*n* = 64)	*p*-Value
	Median (Min–Max)	Median (Min–Max)	
Views	968 (59–392,216)	629 (12–596,755)	0.377
Viewing rate	671.5 (36.7–121,054.3)	358.2 (5.9–375,317.6)	0.434
Likes	21 (2–2819)	21 (1–5255)	0.480
Interaction index	1.4 (0.4–32.5)	2.9 (0.1–1596.8)	0.012 *
GQS Score	2 (1–4)	3 (1–5)	0.046 *
Video score	5 (3–13)	7 (2–16)	0.042 *
DISCERN score	1 (1–4)	2 (1–5)	0.001 *

GOS, global quality scale; Mann–Whitney test; * *p*-value < 0.05.

## Data Availability

The raw data are available from the authors to any author who wishes to collaborate with us.
